# Effect of Soil Management and Training System on Negroamaro Wine Aroma

**DOI:** 10.3390/foods10020454

**Published:** 2021-02-19

**Authors:** Antonio Coletta, Aline Theodoro Toci, Sandra Pati, Giuseppe Ferrara, Francesco Grieco, Maria Tufariello, Pasquale Crupi

**Affiliations:** 1 Council for Agricultural Research and Economics—Research Center for Viticulture and Enology (CREA-VE), Via Casamassima 148, 70010 Turi, Italy; antonio.coletta@crea.gov.it; 2 Environmental and Food Interdisciplinary Studies Laboratory (LEIMAA), Latin American Institute of Life and Nature Sciences (ILACVN), Federal University of Latin American Integration (UNILA), Av. Tancredo Neves, 6731–Bl. 6, Room 8, 85867-970 Foz do Iguaçu, PR, Brazil; aline.toci@unila.edu.br; 3 Department of the Sciences of Agriculture, Food and Environment, University of Foggia, 71122 Foggia, Italy; s.pati@unifg.it; 4 Department of Soil, Plant and Food Sciences, University of Bari “Aldo Moro”, Via G. Amendola 165/A, 70126 Bari, Italy; giuseppe.ferrara@uniba.it; 5 National Research Council-Institute of Sciences of Food Production (CNR-ISPA), Via Prov. Lecce-Monteroni, 73100 Lecce, Italy; francesco.grieco@ispa.cnr.it; 6 Interdisciplinary Department of Medicine, University of Bari “Aldo Moro”, Piazza Giulio Cesare 11, 70124 Bari, Italy

**Keywords:** ethyl esters, alcohols, carboxylic acids, PCA, red wine

## Abstract

This study aimed to assess the impact of two soil managements and training systems on yield and wine aroma compounds of Negroamaro variety grown in a warm climate region (southern Italy). Cover crop (CC) and soil tillage (ST) as soil management, whilst bilateral Guyot (BG) and monolateral Guyot (MG) as training systems were compared. Free and bound volatile fractions were evaluated by GC-MS. ST and CC as well as BG and MG significantly affected yield parameters. In particular, yield was higher in ST and BG than in CC and MG, respectively; moreover, it was found to be positively influenced by interaction between BG and ST. Regarding aroma compounds, significant interactions between soil management and training system factors were observed. In case of free volatiles, the most positive interaction was found between BG and ST, whereas, for bound volatiles, the best interaction was represented by MG with both soil tillage and cover crop. Vine leaf area and development over vine growth stages along with water stress levels played an important role in determining the aroma profile as well as yield parameters. In conclusion, the training system significantly interacted with soil management and affected most of important aroma compounds in Negroamaro wine.

## 1. Introduction

Aroma plays a key role in determining the most important quality attributes of wine [1]. Wine aroma can be classified according to its origin or sequence of wine production into primary (i.e., varietal compounds such monoterpenols, thiols, or norisoprenoids derived directly from grapes), secondary (i.e., pre-fermentative and fermentative compounds) and post-fermentative aroma [2,3,4]. In particular, volatile compounds (such as esters, alcohols, and carboxylic acids) formed during alcoholic fermentation will quantitatively represent most of the aroma constituents, especially in the case of non-aromatic grape vines (*Vitis vinifera* L.) [5,6,7].

As the wine aroma is generally influenced by the berry composition, it can be affected by several factors including variety, pedoclimatic condition, ripeness [8,9], and also agronomical practices, such as soil management and training system [10]. Moreover, the grape varieties have not shown univocal response to viticulture practices and have proved a different adaptation to training systems [11]. However, to date, although the influence of the variety on the wine aroma has been sufficiently investigated, only few studies have been reported on the effects of viticulture practices (i.e., soil management and training system) and even less of their interaction, on the volatile profile and sensory properties of the resulting wines [12,13].

Nowadays, soil tillage is still considered a rather controversial technique. Indeed, while short-term vineyard productivity is usually greater with cultivation than with sod management, long-term cultivation in vineyards has been recognized as a yield-reducing factor. This trend has been attributed to decreased organic matter and nitrogen supply and to reduced soil permeability associated with continuous cultivation [14]. By contrast, cover crop is a technique that improves the soil structure, preventing the soil erosion and limiting the weeds development that can cause a significant reduction in vine growth and yields. Moreover, the cover crop contributes in avoiding the use of herbicides and helping the adoption of organic management of vineyards [15]. Vineyard floor management may contribute in improving overall wine quality, including its aroma profile [16,17]. Indeed, in a recent study aimed to determine the influence of different cover crops and soil tillage on the wine volatile compounds of a Spanish red cultivar, it has been shown how wines from the cover crop treatments tended to have higher concentrations of ethyl esters, volatile fatty acids, and free terpenes than wines from the soil tillage control under that humid climate condition [18]. Training system is an important factor to manage canopy, which plays a fundamental role both in light energy capture via photosynthesis and in water use, as it regulates transpiration and microclimate of ripening grapes, thus affecting yield and quality. Vertical training system shows the vine canopy, extending from the trunk along wires, managed in monolateral or bilateral canes which can be fixed (cordon) or renewable (Guyot). Thus, training system management, determining a different exposure to light, distribution. and orientation of foliage within canopy, vine density and size [5], can influence the berry composition, including the volatile profile and, consequently, the wine aroma [19,20].

Therefore, starting from previous results in the field [12], this work aimed to deepen the effects of cover crop and soil tillage on aroma profile of Negroamaro wine, which is appreciated for its typical intense ruby red color, great body, and fruity note. In particular, the influence exerted by the interaction between soil management (cover crop vs. soil tillage) and training system (monolateral vs. bilateral Guyot) on volatile compounds profile and content was carefully investigated.

## 2. Materials and Methods

### 2.1. Plant Material and Experimental Design

The research was conducted in two consecutive years (2011 and 2012) on berries obtained from an experimental 8-year-old Negroamaro vineyard located in Puglia region (Southern Italy) and grafted on the “SO4” rootstock. The vines were spaced 2.5 m × 1.1 m between and along the rows, respectively, and pruned to 16 buds per vine. Two soil managements were considered: soil tillage (ST) and cover crop (CC), consisting in a mix of 20% *Festuca rubra* L., 20% *Festuca ovina* L., and 60% *Trifolium subterraneum* L.; moreover, two training systems, monolateral (MG) and bilateral (BG) Guyot, were employed. Three replicates, each including 12 rows of 100 vines, were arranged in a split plot experimental design with soil management factor as main plot and training system as subplot. The main plots (ST and CC) were made of 12 rows of 100 vines each, and, within each main plot, the subplots (MG and BG) were made of 6 rows of 50 vines each (Figure S1—Supplementary Materials). The central 4 rows and 26 vines along the rows of the subplot, which allow to obtain 120–130 kg berries, were used for data collection. Vineyards were irrigated according to a controlled water deficit (CWD), which counterbalanced 48% of crop evapotranspiration corresponding to medium water stress. The seasonal irrigation volume, corresponding to 1084 m^3^/ha (108 mm/ha), was managed by a localized irrigation system made by drip lines of 1.6 L/h pressure compensated drip emitters placed between the vines at a distance of 1 m from each other. Starting 10 days after the beginning of veráison (at 10% of berry softening) and until harvest, vineyards were irrigated 5 times and the interval between irrigation cycles was approximately 10 days in each season. A volume of 216 m^3^/ha of water was given in each of the irrigation stage scheduled. The harvest time was 10th October for CC and MG treatments and 17th for ST and BG treatments in 2011, and 5th October for CC and MG treatments and 12th for ST and BG treatments in 2012.

### 2.2. Leaf Area

At flowering and harvest, in each treatment nine measurements of vine leaf area (three vines per replicate) were carried out for each treatment, multiplying the mean shoot numbers per vine by mean shoot leaf area. Mean shoot leaf area was calculated multiplying the mean leaf area (largest + smallest/2) by the number of leaves of the shoot [21]. The smallest and largest leaf area per shoot were determined using a Li-3100C A “leaf area meter” (LI—COR Inc., Lincoln, NE, USA) on 12 shoots per treatment (sampling two representative shoots on six different vines per replication). The same shoots the mean number of leaves per shoot was calculated on the same shoots. Shoot leaf area included primary shoot leaf area and lateral shoot leaf area, which were both calculated applying the same method of calculation.

### 2.3. Plant Water Status (ψ^stem^)

From the beginning of June till the harvest time, the midday stem water potential *ψ_stem_* (MPa) was measured. Before the irrigation period, coinciding with the pre-flowering/berry-set stage (pF-B), it was measured on 1 and 30 May. Then, *ψ_stem_* was measured seven days after each irrigation stage (10 and 20 July, 20 August and 15 September). *ψ_stem_* measurements were performed on three different vines per treatment and on three mature leaves for each vine by a model 600-pressure chamber instrument (PMS Instrument Company, Albany, NY, USA), during the steady period of the water potential diurnal curve (from 11.00 am to 14.00 pm). The used leaves were previously bagged with plastic sheets covered by aluminum foil at least 1 h before the measurement.

### 2.4. Yield Components and Fruit Composition

The yield (kg/vine) was determined at harvesting by averaging nine vines production per replicate. The cluster weight was determined on the same vines by weighing three clusters per vine. The cluster number was calculated dividing the yield by cluster weight for each vine. The mean berry weight was determined sampling 50 berries per replicate from the same clusters utilized for cluster weight determination. Then, for each replicate, the pulp of berries was pressed, and the juice was analyzed for total soluble solids (TSS, Brix), pH, and titratable acidity (TA, g/L tartaric acid), together with tartaric, malic, and citric acids determination according to EEC 2676 standard procedure [22].

### 2.5. Winemaking

From each independent replicate of field treatment (ST × MG; ST × BG; CC × MG; CC × BG), about 80 kg of berries were picked at “technological maturity”, corresponding to a total soluble solids (TSS) value of 21–22 Brix. Berries were previously destemmed and then vinified. All vinifications (4 × 3 replicates) were performed in 100-kg-capacity stainless steel micro-vinificators. Briefly, the berries were crushed, and 80 mg/L of potassium metabisulfite was added. The maceration was carried out for 24 h at the cellar temperature of 15 °C and three pumping over were carried out during the above period. After maceration, berries were pressed (1.6 atm) and the solid parts were removed. Then, 25 g/HL of hydrated yeasts (*Saccharomyces cerevisiae*, Zymasil, AEB) was inoculated and 25 g/HL of a prepared to nutritional yeast (ammonium sulfate, ammonium phosphate dibasic, filter aid, and vitamin B1, Enovit, AEB) was added. After 24 h, Lalvin 31 (MBR) (Lallemand Inc, Verona, Italy) *Oenococcus oeni* single-strain culture (2 × 10^7^ cfu/mL) was inoculated prior re-hydrated in water at 20 °C for 15 min and then, the fermentation temperature was maintained below 25 °C. Fermentations were monitored by measuring the sample specific density and malic acid content from the inoculation moment and every 12 h until a constant specific value was reached. At the end of alcoholic and malolactic fermentations and after static decantation, the wines, which showed same chemical characteristics (Table S1—Supplementary Materials), were racked, added with 40 mg/L of potassium metabisulfite, bottled into dark green Bordeaux bottles, and stored at 10–12 °C for 6 months.

### 2.6. Wine Volatile Compounds Extraction and GC/MS Analysis

Free and glycoside bound volatiles (FV and BV, respectively) were recovered from the wines by solid phase extraction (SPE), according to Toci et al. [12], in triplicate. Briefly, 125 mL of wine, containing 60 µL of butylated hydroxyanisole (12.66 mg/mL in ethanol) and 1.2 µL of internal standard (2-octanol), were loaded onto a SPE cartridge (STRATA-X, Polymeric Reversed Phase, 33 µm, Phenomenex). FV compounds were eluted by 5 mL of dichloromethane and collected in 7 mL amber vials. Five mL of methanol were then applied to the same cartridge to elute the more polar fractions, containing the BV compounds. The FV solvent was evaporated by N_2_ flow until 0.5 mL, whilst the BV fraction was firstly dried on rotating evaporator (Buchi Italia s.r.l., Cornaredo, Italy) at 40 °C and then solubilized in 5 mL of citrate-phosphate buffer at pH 5 with addition of 200 mg of LISAROM enzyme with strong secondary glycosidase activity (Enolife s.r.l., Montemesola, Italy), to conduct enzymatic hydrolysis.

The solutions were placed in a water bath for 24 h at 40 °C. The digests were then centrifuged (3000 rpm for 2 min), and the supernatants were loaded onto new SPE STRATA-X cartridges. BV compounds were eluted by 5 mL of dichloromethane, collected in 7 mL amber vials, and the solvent evaporated by N_2_ flow until 0.5 mL. Both FV and BV were stored at −20 °C until further analyses. An Agilent 6890 GC equipped with an Agilent 5975 mass spectrometer (Wilmington, USA) and a DB-Wax (60 m × 0.25 mm i.d.; 0.5 µm film thickness) column from J &W Scientific Inc. (Folsom, CA, USA) were used. The chromatographic conditions were injection mode: split 1:20, injection temperature: 250 °C; temperature setting: 40 °C (5 min) to 200 °C (15 min) at 2 °C/min, to 250 °C at 1 °C/min, detector temperature: 280 °C, carrier gas: helium, flow: 1.0 mL/min. The mass spectrometer operated in the electron impact mode (ionization energy, 70 eV), using a mass range of *m/z* 28–300 and a scan interval of 1.0 s.

### 2.7. Data Processing

Total ion chromatograms were processed using the automated data processing software MSD ChemStation (Agilent Technologies, Wilmington, NC, USA). NIST-2004 spectral library, Kovats Index, and reference standards, when available, were used for peak identification. While, the quantification was performed by the internal standard method, using calibration curves (from 4 to 7 levels of magnitude for covering the normal range of occurrence of most compounds in wine) for 20 volatile compound standards (Fisher Scientific Italia, Rodano, Italy) belonging to the main volatile classes identified. The relationship between the signal (ion peak area normalized by the internal standard, 2-octanol) and the concentration is linear, and the squared regression coefficients (R^2^) are higher than 0.9 (Table S2—Supplementary Materials). Because of the lack of commercial standards, the other identified compounds were quantified, according to their structural characteristics, as equivalents of the compounds used as calibrants.

### 2.8. Statistical Analysis

Values were shown as means over the 2 years. On the collected data, a two way analysis of variance (ANOVA) was carried out by STATISTICA software v. 8.0 (StatSoft Inc., Tulsa, OK, USA) to analyze the effects of the two soil managements and training systems, separately, and together with their significant interactions. F test was used to compare the means within the soil management and training system. The Fisher LSD multiple range test was used to compare the interaction significant effects. Principal Component Analysis (PCA) was finally performed in order to describe clustering effect of aroma compounds and relationship with different soil management and training system.

## 3. Results and Discussion

### 3.1. Yield Parameters and Fruit Composition

**Figure 1:**
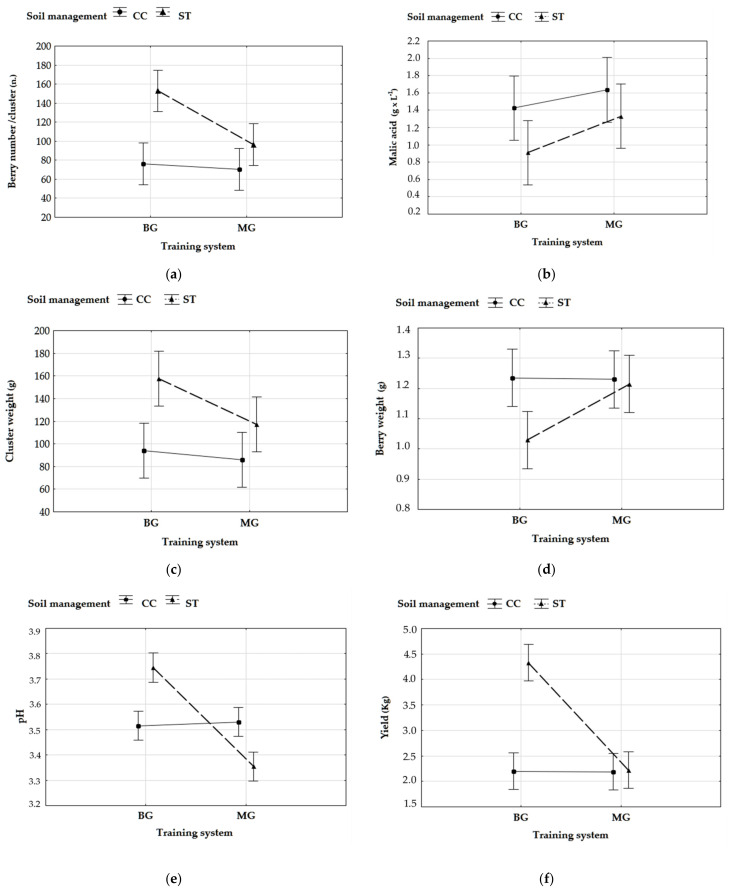
Significant interactions between soil management and training systems treatments on (**a**) berries number/cluster, (**b**) malic acid (g/L), (**c**) cluster weight (g), (**d**) berry weight (g), (**e**) pH, and (**f**) yield (kg/vine), ST: Soil tillage; CC: Cover crop. MG: monolateral Guyot; BG: bilateral Guyot.

Soil management clearly affected the yield (*p* < 0.01), that was lower for CC (2.19 kg/vine) than ST treatment (3.27 kg/vine) due to the competition between vines and CC, as reported by other authors [12,23]. On the other side, the improvement exerted by ST on the yield was mainly due to the significant effect on cluster weight (around +53%), whereas cluster number was not statistically different, as expected (Table 1). As regards the training system, BG, allowing a better light distribution within the shoots, gave rise to a significantly (*p* < 0.01) higher yield (3.17 kg/vine) than MG (2.29 kg/vine) thanks to both berries weight and berries number per cluster (Table 1), confirming the findings of previous studies [24,25]. Moreover, the bilateral system positively interacted with ST, leading to the highest cluster weight and, consequently, yield (Figure 1c,f).

With regard to berry characteristics, the weight was affected by training system (*p* < 0.05) and almost 16% higher values were found in MG berries. Whilst, no significant influence of soil management factor was registered, even though the lowest berry weight (1.03 g) was found in combined BG × ST samples (Table 1; Figure 1d). A strong change in berries number was induced by both the factors (*p* < 0.01), in particular the number of berries was more abundant (about 150) in the clusters from vines experimenting ST and BG simultaneously (Figure 1a). This behavior could be attributed to the reduced vine water stress occurred either in ST (−1.08 MPa) or in BG (−1.07 MPa) over the pF-B stage.

TSS and TA were influenced neither by soil management nor training system as well as by interaction of the two factors (Table 1), in accordance with other studies confirming that, at harvesting, the macro-constituents are generally not affected by soil management and training system [12,26,27]. In addition, berry sugar concentration is known to be a relatively stable trait for a given cultivar, being less responsive to environmental conditions and viticulture practices than anthocyanins [28] and organic acids [29], and only malic acid varied depending on the soil management (more concentrated in CC) and training system (more concentrated in MG) (Table 1; Figure 1b). These differences were ascribable to those conditions (such as soil tillage and bilateral systems) favoring the reduction of canopy density and cluster shading and, consequently, the increase of temperature in the cluster zone. This caused better leaf area to fruit ratio registered in BG and ST, which led to stronger malic acid degradation along the berry ripening stage [5]. Finally, pH values were very similar in ST and CC samples but they significantly differed in MG and BG ones (Table 1; Figure 1e).

### 3.2. Leaf Area and Leaf Area to Crop Ratio

The vine leaf area is an important factor variating mostly near the flowering stage and affecting both yield and quality. As reported in Table 2, it was larger in ST than in CC both at flowering (4.3 vs. 2.4 m^2^, respectively) and harvesting (5.8 vs. 4.5 m^2^, respectively) time. This effect can be explained arguing that CC strongly competed with vine leaf growth in the first stages of vine development, in particular from flowering to berry-set. Afterwards, as the cycle of CC ended up (particularly for *T. subterraneum*), the competition diminished. This variation in leaf area, depending on soil management, obviously conditioned *ψ_stem_* during the ripening stage (Table 3). In fact, in CC management the presence of the floor vegetation reduced the evaporation from the soil and the smaller leaf area of the vines also reduced transpiration, thus leading to an overall reduction of water consumption. In contrast, ST induced an increase of soil evaporation due to tillage effect and a higher transpiration demand due to the larger leaf area of the tilled vines. Accordingly, lower *ψ_stem_* with higher water stress were registered both at berry set (pF-B) in CC and at ripening stage (V-H) in ST (Table 3). With regard to the training systems, a small difference in leaf area was only found at flowering stage (Table 2), because of better growth conditions in BG. Indeed, the shoots showed a homogeneous growth in BG plot because of the lower number of nodes along the cane and their better spatial and light distribution due to the opposite direction of the canes [30]. The initial leaf area advantage of BG was almost disappeared at harvesting (Table 2), probably because BG had a higher water transpiration due to both the wider leaf area and higher exposition of leaves to light during the season.

About the leaf area to crop ratio, values ranging from 8 to 12 (cm^2^/g) are considered the hallmark of a balanced vine [31]. This ratio could be acceptable for wine grapes even when lower than 8 or higher than 14 (cm^2^/g). Then, for vines grown with vertical shoot (such as BG and MG), lower and upper limits depend on the growing region climate; in Mediterranean regions, the leaf area optimal value can vary from 15 to 20 cm^2^/g [32]. In our case, training system and soil management differently affected the leaf area to crop ratio during the growth stages (Table 2). At harvesting MG, allocating the vines canopy in a compact body, comprising all the shoots, and decreasing the photosynthetic efficiency, showed the highest ratio (23.15). In ST and BG leaf area to fruit weight ratios were very close to the optimal level ranging from 17.8 to 15.8, respectively. These more balanced ratios were due to a very high yield per vine rather than a lower leaf area (Table 1 and Table 2). In particular, BG clearly appeared as the best condition for vines production as it allowed, besides of the best ratio (15.83), the best balanced canopy as foliar surface did not overcome 5.02 m^2^/vine. In addition, BG confirmed these positive aspects allowing very interesting yield results (3.17 kg/vine) along with a valuable TSS value (22.07 Brix).

### 3.3. Volatile Compounds

A total of 45 compounds were identified and quantified by SPE-GC-MS analyses in free and enzymatic hydrolyzed fractions from Negroamaro wines. According to their functional groups, they are classified into esters, carboxylic acids, alcohols, phenolics, acetamides, sulfurs, and carbonyl compounds both in free and bound forms; bound compounds were almost 10-fold less concentrated than the free ones, thus revealing an overall low aroma potential (glycoside volatile compounds) of Negroamaro wines (Table 4 and Table 5). The highest free volatiles content was determined in ST and BG (62,000 ± 9000 and 64,000 ± 9000 g/L) compared to CC and MG wines (59,000 ± 6000 and 57,000 ± 8000 g/L). Furthermore, a significant interaction (*p* < 0.001) ST × BG was also found for free volatiles (Table 4). As reported elsewhere [33], the observed variations may be caused by the different yields and, especially, the different clusters exposure to light in the two treatments (Table 2 and Table 3). A better exposure of bunches to light was achieved by ST and BG because the former lowers the canopy density due to a higher water competition between vines (Table 3), while the latter reduces the shade on bunches as typically observed in training systems with divided canopies [12,25]. However, it is worth pointing out that in other climate conditions (i.e., rainy temperate climate region) CC caused a stronger water stress limiting the canopy of vines and favoring aroma production [17].

Several authors also found a higher content of glycoside precursors in wines from clusters growth with increasing light exposure [2,13]. Surprisingly, in our study, these compounds resulted more concentrated in MG wines, despite a decreased light exposure of clusters. However, since Ugliano et al. have suggested that the hydrolytic β-glucosidase activity of some *Oenococcus oeni* strains (i.e., Lalvin 31-MRB) rapidly decreased at lower pH [34], it could be inferred that the low pH of MG berries (and consequently must) did not favor the de-glycosylation of aroma compounds during the malolactic fermentation, motivating the higher levels of glycosides in MG wines (Table 1).

Because of the synergic and antagonistic effects of the compounds forming the wine aroma [35], a comparison of each chemical group among wines was carried out for better evaluating the differences in Negroamaro wines as affected by agricultural practices in vineyard. Higher alcohols are synthesized by yeast during alcoholic fermentation mainly through the catabolism of the corresponding amino acids whose content in berries can be affected by viticultural practices [36]. Six alcohols were identified in the free volatile fractions and their content was strongly affected by soil management in accordance with our previous finding [12]. The most sun exposure of berries grown under ST condition should explain the major alcohols concentration in the related wines (Table 4), even though contrasting results merged in literature concerning the impact of light on wine higher alcohols [37]. Instead, no difference in alcohols content was generally observed between the two guyot systems, in disagreement with other reports showing how vertical training systems (similar to BG) favored the accumulation of higher alcohols [25,26]. However, it is worth noting the significantly (*p* < 0.05) positive interaction ST × BG on the concentration of the detected alcohols (except for 3-methyl-1-pentanol); in particular, the highest amount of 2-phenyl-ethanol (> 3,0000 µg/L) was revealed in samples derived from berries experimenting ST and BG contemporarily. Only 3-methyl-butanol and 2-phenyl-ethanol were identified in glycosylated fractions, with the highest values registered in CC × MG and ST × MG samples, respectively (Table 5).

Ethyl esters are one of the most important group of volatiles, contributing to the typical fruity aroma of young wines [12]. They are produced enzymatically during fermentation and from ethanolysis of acetyl-CoA during fatty acids synthesis or degradation; therefore, their concentration depends not only on enological factors but also on viticultural parameters influencing the berry composition [5]. For instance, several reports in literature have demonstrated that a greater cluster exposure to sunlight increases the content of wine esters [12,38]. Ethyl esters concentrations (Table 4) were, generally, not affected by either soil management or training system factors, singularly. However, interestingly, they were significantly influenced by the interaction of the two factors; the highest value of these compounds (5500 µg/L) was found in ST × BG samples, confirming that wines made from berries from better-lit environments performed better with regard to ethyl esters content [20]. Only two esters were identified in glycosylated fractions; in particular, just the concentration of diethyl succinate significantly changed with soil management and training system as well as their interaction, showing the best increase in CC × MG wines (Table 5).

Ten volatile fatty acids were identified, among which hexanoic and octanoic acids were the major compounds in the samples (Table 4). Overall, they are not associated with the aromatic wine quality, having high odor thresholds [12]; anyway, these compounds in some case (e.g., 2-methyl propanoic acid and hexanoic acid) can play an indirect role in the complexity of the aroma contrasting the hydrolysis of the corresponding esters [39]. A significant increase of free fatty acids content was related with those conditions improving berries exposure to light, namely, ST and BG as well as their interaction ST × BG; indeed, the highest concentration (~ 5400 µg/L) of acids was determined in ST × BG Negramaro wines (Table 4). Literature results are controversial, in the sense that some studies claimed the increment of volatile fatty acids with sunlight exposure [38,40], while others reported no effect [41,42] or even their decrease [43]. Moreover, fatty acid volatile compounds have been described to be affected by training systems that provide different growing conditions and light exposure, but the influence varied in different wine regions [19,33]. Seven compounds were revealed as glycosylated fatty acids, whose concentration (ranging from 1310 to 1700 µg/L) strongly depended on the two growing factors. However, their greatest values were found in CC and MG samples (Table 5).

Regarding the other compounds, 2,6-dimethoxy-phenol and carbonyl compounds (e.g., 2-octanone) were found in both free and glycosylated fractions of the analyzed wines (Table 4 and Table 5). In line with our previous report [12], higher contents of free and glycosylated 2,6-dimethoxy-phenol were determined in ST (104 ± 9 µg/L) and CC (36.3 ± 1.1 µg/L) wines, respectively; conversely, carbonyl compounds were more concentrated in CC wine (290 ± 40 µg/L). Finally, no significant difference was observed in phenolics and ketones both in free and bound forms of MG and BG wines. N-(2-phenylethyl)-acetamide and N-(3-methylbutyl)-acetamide were detected in the free volatile fraction, with values ranging from 800 to 1130 µg/L. Their total content was strongly (*p* < 0.001) increased by ST treatment, while neither significant influence of training system factor nor significant interaction was recorded (Table 4). Moreover, in glycosylated fraction, N-(2-phenylethyl)-acetamide was just found in ST and MG wines (Table 5). Two sulfur compounds were only identified in the free volatile fractions, in particular the highest concentration of 3-methylthio-1-propanol (having very unpleasant aroma, which resemble cooked cauliflower) was present in CC × MG wines (Table 4).

Overall, it can be summarized that ST positively affected free volatiles content. As reported by Monteiro and Lopes [23] the higher water stress level registered in ST with respect to CC, especially during VH growth stage (Table 3), influenced the concentrations of free volatiles. The water stress by ST contributed to reduce the canopy growth until veráison and to achieve at harvest a leaf area to fruit ratio closer to the optimal value of 12–15 cm^2^/g than CC (Table 2) [32].

Differently by soil managements, BG and MG induced neither significant differences in plant water conditions (Table 3) nor differences in leaf area. Nevertheless, training systems affected the content of total free volatile aroma compounds (Table 5) in a stronger way than soil managements. Finally, concerning the training systems comparison it has to take into account the different pH values (Table 3) that in case of MG was more acid (3.34) than BG (3.63) thus contributing to favor glycolsylated compound content at the expense of free forms (as inferred in the Section 3.3).

**Figure 2:**
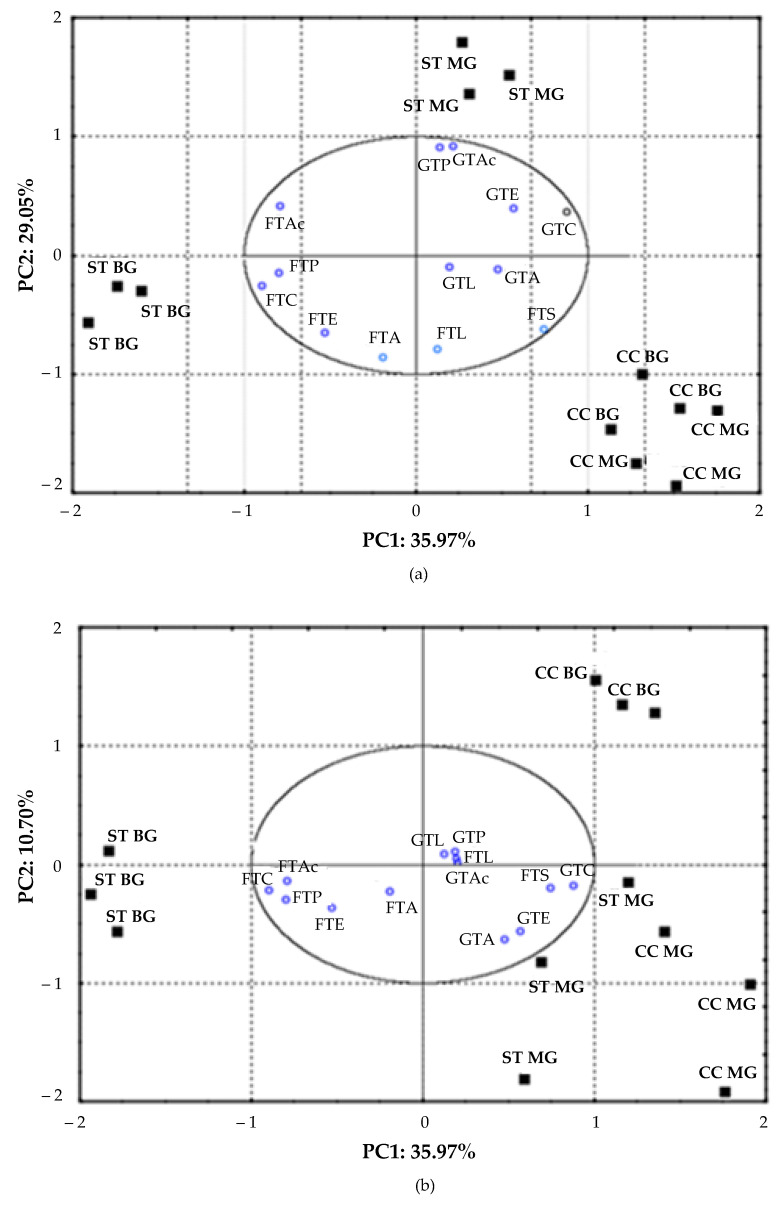
Principal component analyses (PCA) of free and glycosylate aroma compounds are reported showing scores plot and projection of variables on (**a**) PC 1 vs. PC 2 and (**b**) PC 1 *vs* PC 3 components. Variables correspond to total free and glycosylated alcohols (FTA and GTA), esters (FTE and GTE), carboxylic acids (FTC and GTC), phenols (FTP and GTP), acetamides (FTAc and GTAc), sulfurs (FTS), and lactones (FTL and GTL). Cases grouping was calculated considering each soil tillage (ST), cover crop (CC), monolateral Guyot (MG), and bilateral Guyot (BG) interactions.

To achieve a global and complete overview of the interactive effect of the two factors in determining a discriminatory output, PCA was carried out on volatiles amounts (variables) calculated for each soil management and training system interaction (cases). In particular, total free and glycosylated groups of compounds that exhibited significant difference from the two ‒way ANOVA analyses were considered. Overall, PC1, PC2, and PC3 explained 75.72% of total variance (Figure 2). ST × BG wines, mainly characterized by free total alcohols (FTA), carboxyl acids (FTC), esters (FTE), and acetamides (FTAc) having the greatest factor loadings (>|0.9|) on PC1, were clearly separated from CC × BG and CC × MG wines. Moreover, total glycosylated alcohols (GTA), esters (GTE), and acetamides (GTAc), which were positively related to PC2, allowed to cluster ST × MG samples (Figure 2a). This finding means that in ST condition the employed training system played a key role in differentiating aroma composition as free (in BG case) or glycosylated compounds (in MG case). A supplement separation between CC × BG and CC × MG was observed along PC3 (responsible for 10.70% of explained total variance), which confirmed how MG training system would favor the formation of glycosides in CC condition, too (Figure 2b).

## 4. Conclusions

This study reported how fermentation aroma compounds in Negroamaro wines were affected both by soil management and training system. In particular, ST management and BG system showed to preferentially favor free aroma compounds formation. Whilst CC and MG conditions led to an increase of bound compounds in wines. This finding seemed related to the fact that the studied soil managements and training systems differently conditioned water availability and leaf area to fruit ratio as well as vine microclimate starting from the bud-break stage. Finally, yield parameters were also taken into account to completely assess the productive consequences of the agronomic practices on the vineyard. In conclusion, this work confirmed the importance of a variety-dependent investigation about the influence of agronomic practices for giving new insight which might help winemakers to better control the wine quality.

## Figures and Tables

**Table 1:** Effects of soil management and training systems on yield components and fruit composition.

Variables	Factors					
	Soil Management			Training System		
	ST	CC	S	MG	BG	S
Yield (kg/vine)	3.3 ± 1.2	2.2 ± 0.7	**	2.3 ± 0.7	3.2 ± 1.2	**
Cluster weight (g)	140 ± 20	90 ± 14	**	100 ± 20	130 ± 40	n.s.
Cluster number	23 ± 5	25 ± 4	n.s.	20 ± 5	28 ± 2	*
Berry weight (g)	1.12 ± 0.11	1.23 ± 0.09	n.s.	1.26 ± 0.05	1.09 ± 0.11	*
Number of berries	120 ± 30	77 ± 13	**	83 ± 18	110 ± 30	**
TSS (Brix)	21.2 ± 0.5	22.2 ± 0.9	n.s.	21.3 ± 0.9	22.1 ± 0.4	n.s.
TA (g/L)	5.4 ± 0.4	5.3 ± 0.3	n.s.	5.2 ± 0.4	5.60 ± 0.15	n.s.
Citric acid (g/L)	0.23 ± 0.08	0.20 ± 0.09	n.s.	0.19 ± 0.06	0.14 ± 0.05	n.s.
Malic acid (g/L)	1.1 ± 0.3	1.53 ± 0.14	*	1.5 ± 0.3	1.1 ± 0.2	*
Tartaric acid (g/L)	5.1 ± 0.7	5.2 ± 0.3	n.s.	5.1 ± 0.6	5.2 ± 0.5	n.s.
pH	3.55 ± 0.15	3.52 ± 0.06	n.s.	3.34 ± 0.10	3.63 ± 0.14	*

*, **, n.s.: significant interaction effects are shown, they are significant at *p* < 0.05, *p* < 0.01, *p* < 0.001, or not significant, respectively. ST: Soil tillage; CC: Cover crop; MG: monolateral Guyot; BG: bilateral Guyot; S: significance.

**Table 2:** Vine total leaf area (m^2^) at berry-set and harvesting stage in the soil management and training system with calculated leaf area to fruit ratio (cm^2^/g) at harvesting.

Factors	Growth Stage		
	Berry-set	Harvesting	
	Total Leaf Area Per Vine	Total Leaf Area Per Vine	Leaf Area to Fruit Ratio
**Soil management**			
ST	4.3 ± 1.2	5.8 ± 1.7	18 ± 6
CC	2.4 ± 0.8	4.5 ± 1.3	20 ± 7
S	**	*	*
**Training systems**			
MG	3.4 ± 1.2	5.3 ± 1.9	23 ± 9
BG	4.3 ± 1.1	5.0 ± 1.4	16 ± 5
S	*	n.s.	**
Interaction	n.s.	n.s.	n.s.

*, **, n.s.: significant interaction effects are shown, they are significant at *p* < 0.05, *p* < 0.01, *p* < 0.001, or not significant, respectively. ST: Soil tillage; CC: Cover crop; MG: monolateral Guyot; BG: bilateral Guyot; S: significance.

**Table 3:** Averaged water stem potentials *ψ_stem_* (MPa) over all the pre-flowering/harvesting (pF-H), pre-flowering/berry-set (pF-B), berry-set/veráison (Bs-V) and veráison-harvesting (V-H) stages, of Negroamaro berry growth in soil management and training system treatments.

	Growth Cycle			
	pF-H	pF-B	Bs-V	V-H
**Soil management ^a^**				
ST	−1.18 ± 0,08	−1.08 ± 0.01	−1.25 ± 0.08	−1.21 ± 0.14
CC	−1.09 ± 0.10	−1.18 ± 0.02	−1.03 ± 0.11	−1.06 ± 0.09
S	n.s.	*	*	**
**Training system ^b^**				
MG	−1.14 ± 0,08	−1.19 ± 0.01	−1.09 ± 0.08	−1.14 ± 0.19
BG	−1.13 ± 0.10	−1.07 ± 0.01	−1.19 ± 0.11	−1.13 ± 0.18
S	n.s.	*	*	n.s.
Interaction	n.s.	n.s.	n.s.	n.s.

*,**, n.s.: significant interaction effects are shown, they are significant at *p* < 0.05, *p* < 0.01, *p* < 0.005, or not significant, respectively. ^a^ ST: Soil tillage; CC: Cover crop; ^b^ MG: monolateral Guyot; BG: bilateral Guyot; S: significance.

**Table 4:** Concentration (µg/L—means values and standard deviation) of single free volatile compounds identified in the Negroamaro wines from the two soil managements and training systems by SPE-GC-MS.

Compounds	Factors						
	Soil Management			Training System			Interaction
	ST	CC	S	MG	BG	S	S
Esters							
Ethylbutanoate	280 ± 110	370 ± 180	**	340 ± 190	310 ± 100	n.s.	**
Ethyl 3-methylbutanoate (isoamyl acetate)	380 ± 60	250 ± 120	*	310 ± 110	320 ± 130	n.s.	n.s.
Ethyl hexanoate (ethyl caproate)	140 ± 30	110 ± 60	n.s.	130 ± 40	110 ± 50	n.s.	**
Ethyl 2-hydroxypropanoate	220 ± 60	230 ± 50	n.s.	240 ± 60	230 ± 60	n.s.	**
Ethyl 3-hydroxybutanoate	n.d.	6.3 ± 1.1	***	6.3 ± 1.1	n.d.	***	***
Diethyl butanedioate (diethyl succinate)	570 ± 80	530 ± 80	n.s.	550 ± 60	550 ± 100	n.s.	***
Ethyl 4,4-ethoxyhydroxybutanoate	3200 ± 1700	3300 ± 900	n.s.	2800 ± 1300	3600 ± 1300	*	***
Total	5000 ± 2000	4800 ± 1400	n.s.	4500 ± 1700	5200 ± 1700	n.s.	***
Carboxylic Acids							
Acetic acid	670 ± 140	200 ± 30	***	230 ± 40	630 ± 180	***	***
Propanoic acid	4.3 ± 0.4	2.6 ± 0.3	**	13 ± 4	44 ± 7	***	**
2-methyl propanoic acid (isobutyric acid)	110 ± 40	121 ± 16	**	100 ± 40	130 ± 20	***	***
Butanoic acid	70 ± 40	58 ± 18	n.s.	82 ± 5	48 ± 8	**	**
3-methyl-butanoic acid (isovaleric acid)	280 ± 80	380 ± 40	***	320 ± 120	350 ± 10	*	***
Pentanoic acid (valeric acid)	82 ± 5	53 ± 3	***	73 ± 5	55 ± 7	**	***
Hexanoic acid (caproic acid)	1300 ± 200	1280 ± 80	n.s.	1240 ± 100	1380 ± 160	**	***
Octanoic acid (capric acid)	1700 ± 300	1500 ± 300	n.s.	1500 ± 200	1700 ± 400	n.s.	**
Benzoic acid	250 ± 80	290 ± 150	n.s.	260 ± 170	280 ± 50	n.s.	n.s.
Benzeneacetic acid	290 ± 60	130 ± 30	***	200 ± 50	220 ± 140	n.s.	***
Total	4800 ± 1000	4000 ± 700	*	4100 ± 700	5200 ± 1000	**	***
Alcohols							
2-methyl-propanol	1100 ± 110	560 ± 50	**	550 ± 50	1100 ± 110	***	***
3-methyl-butanol (isoamyl alcohol)	20,000 ± 2700	23,000 ± 1800	**	20,000 ± 4000	22,000 ± 1000	n.s.	**
3-methyl-1-pentanol	27 ± 4	71 ± 19	***	50 ± 30	43 ± 18	n.s.	n.s.
1-hexanol	470 ± 80	420 ± 40	*	450 ± 50	450 ± 100	n.s.	***
3-ethoxy-1-propanol	n.d.	0.27 ± 0.08	***	0.17 ± 0.09	0.10 ± 0.02	**	**
2-phenyl-ethanol (phenylethyl alcohol)	28,000 ± 3000	24,000 ± 2000	*	24,600 ± 1100	27,000 ± 5000	n.s.	*
Total	50,000 ± 6000	48,000 ± 4000	*	46,000 ± 5000	51,000 ± 6000	n.s.	*
Phenolics							
2,6-dimethoxy-phenol (syringol)	104 ± 9	84 ± 5	**	98 ± 13	90 ± 12	n.s.	n.s.
Acetamides							
N-(2-phenylethyl)-acetamide	1030 ± 140	960 ± 200	n.s.	890 ± 110	1130 ± 150	*	n.s.
N-(3-methylbutyl)-acetamide	1090 ± 140	490 ± 140	***	800 ± 300	800 ± 300	n.s.	*
Total	2100 ± 300	1500 ± 300	***	1700 ± 400	1900 ± 400	n.s.	n.s.
Sulfurs							
Dihydro-2-methyl-3-(2H)-thiphenone	n.d.	10 ± 3	***		4.0 ± 1.8	n.s.	n.s.
3-methylthio-1-propanol	11.7 ± 1.0	120 ± 40	***		38 ± 4	***	***
Total	11.7 ± 1.0	130 ± 40	***		42 ± 6	***	***
Ketones Lactones Aldehydes							
2-octanone	154 ± 19	230 ± 30	***		210 ± 50	n.s.	n.s.
Butyrolactone	40 ± 6	36 ± 5	n.s.		48 ± 7	***	***
Benzaldehyde	19 ± 4	23 ± 6	n.s.		21 ± 7	n.s.	n.s.
Total	210 ± 30	290 ± 40	***		280 ± 60	n.s.	*

*, **, ***, n.s.: significant interaction effects are shown, they are significant at *p* < 0.05, *p* < 0.01, *p* < 0.001, or not significant, respectively. ST: Soil tillage; CC: Cover crop; MG: monolateral Guyot, BG: bilateral Guyot; S: significance.

**Table 5:** Concentration (µg/L—means values and standard deviation) of single glycosylate volatile compounds identified in the Negroamaro vines from the two soil managements and training systems by SPE-GC-MS.

Compounds	Factors						
	Soil management			Training system			Interaction
	ST	CC	S	MG	BG	S	S
Esters							
Diethyl butanedioate (diethyl succinate)	64 ± 9	145 ± 14	***	132 ± 11	77 ± 9	***	***
Ethyl 4,4-ethoxyhydroxybutanoate	290 ± 60	210 ± 130	n.s.	310 ± 90	190 ± 70	*	n.s.
Total	350 ± 70	360 ± 140	n.s.	440 ± 100	270 ± 80	**	n.s.
Carboxylic Acids							
Acetic acid	72 ± 9	80 ± 30	n.s.	149 ± 10	n.d.	***	n.s.
Butanoic acid	58 ± 14	n.d.	***	56 ± 12	n.d.	***	***
Pentanoic acid (valeric acid)	61 ± 17	n.d.	***	59 ± 14	n.d.	***	***
Hexanoic acid (caproic acid)	250 ± 30	80 ± 20	***	180 ± 20	150 ± 20	n.s.	***
Octanoic acid (capric acid)	510 ± 50	850 ± 80	***	640 ± 40	720 ± 60	*	**
Benzoic acid	260 ± 40	360 ± 60	**	340 ± 70	280 ± 50	*	n.s.
Benzeneacetic acid	97 ± 12	158 ± 11	***	260 ± 70	n.d.	***	***
Total	1310 ± 170	1500 ± 200	**	1700 ± 200	1150 ± 130	***	**
Alcohols							
3-methyl-butanol (isoamyl alcohol)	90 ± 20	139 ± 120	***	210 ± 80	230 ± 50	***	***
2-phenyl-ethanol (phenylethyl alcohol)	150 ± 50	70 ± 20	***	132 ± 17	90 ± 20	*	***
Total	240 ± 70	210 ± 30	n.s.	340 ± 90	110 ± 30	**	*
Phenolics							
2,6-dimethoxy-phenol (syringol)	15 ± 2	36.3 ± 1.1	**	28 ± 16	24 ± 11	n.s.	n.s.
Acetamides							
N-(2-phenylethyl)-acetamide	3.9 ± 1.4	n.d.	***	4.9 ± 1.2	n.d.	***	***
Ketones							
2-octanone	132 ± 11	130 ± 20	n.s.	142 ± 14	123 ± 14	n.s.	n.s.

*, **, ***, n.s.: significant interaction effects are shown, they are significant at *p* < 0.05, *p* < 0.01, *p* < 0.001, or not significant, respectively. ST: Soil tillage; CC: Cover crop; MG: monolateral Guyot, BG: bilateral Guyot; S: significance.

## Data Availability

The datasets generated for this study are available on request to the corresponding author.

## Supplementary Materials

The following are available online at https://www.mdpi.com/2304-8158/10/2/454/s1, Figure S1: Split plot experimental design with soil management factor as main plot and training system as subplot; Table S1: Chemical characteristics of Negroamaro wines; Table S2: Parameters used for identification and quantitation by SPE-GC-MS of volatile compounds in Negroamaro wines.
