# Supplementary material for: Effect of Soil Management and Training System on Negroamaro Wine Aroma
Source: Foods. 2021 Feb 19;10(2):454. doi: 10.3390/foods10020454 (PMC7922071; doi:10.3390/foods10020454)
Supplement: Supplementary file 1 [file foods-10-00454-s001.pdf]

Figure S1. Split plot experimental design with soil management factor as main plot and training system as subplot

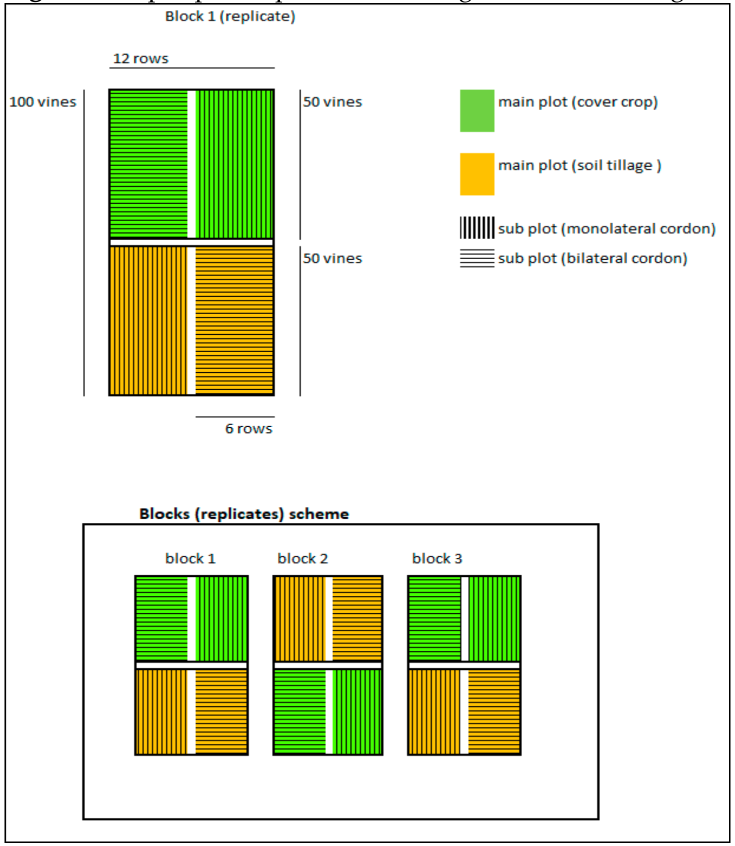

Table S1. Chemical characteristics of Negroamaro wines.

	**E(% *v*/*v*)**	**pH**	**VA (g L^{-1})**	**Total SO_2 (mg L^{-1})**	**Free SO_2 (mg L^{-1})**
Soil management					
CC	14.0 ± 0.7	3.40 ± 0.12	0.25 ± 0.02	80 ± 2	38 ± 3
ST	13.7 ± 0.5	3.45 ± 0.19	0.29 ± 0.04	79 ± 3	36 ± 2
Significance	n.s.	n.s.	n.s.	n.s.	n.s.
Training system					
BG	13.9 ± 0.8	3.35 ± 0.11	0.26 ± 0.04	81 ± 4	36 ± 3
MG	13.6 ± 0.6	3.42 ± 0.13	0.28 ± 0.05	79 ± 2	37 ± 3
Significance	n.s.	n.s.	n.s.	n.s.	n.s.

E, ethanol; TA, titratable acidity as tartaric acid equivalent; VA, volatile acidity as acetic acid equivalent. n.s., not significant.

Table S2. Parameters used for identification and quantitation by SPE-GC-MS of **v**olatile compounds in Negroamaro wines.

No.	Classes	Volatile compounds	ID [a]	LRI [b]	Linear range (µg/L)	Intercept	Slope	R^2	Ion identification
1	Esters	Ethylbutanoate	A	1020	50–650	– 0.003680	0.000370	0.9990	88-71-43
2		Ethyl 3-methylbutanoate (isoamyl acetate)	A	1094	50–650	– 0.000847	0.000380	0.9995	70-55-43
3		Ethylhexanoate (caproate)	A	1218	50–650	– 0.006194	0.000444	0.9973	72-45-30
4		Ethyl 2-hydroxypropanoate	B	1321	-- [c]	--	--	--	75-45
5		Ethyl 3-hydroxybutanoate	B	1499	--	--	--	--	117-87-60
6		Diethylbutanedioate (diethyl succinate)	A	1654	50–650	– 0.005339	0.000810	0.9988	129-101-29
7		Ethyl 4,4-ethoxyhydroxybutanoate	C	2339	--	--	--	--	128-101-73
8	Carboxilic acids	Acetic acid	A	1427	--	--	--	--	60-45-43
9		Propanoic acid	A	1512	200–1000	– 0.001409	0.000221	0.9913	74-57-45
10		2-methyl propanoic acid (isobutiric acid)	A	1541	200–1000	– 0.000804	0.000357	0.9888	73-43
11		Butanoic acid	A	1599	200–1000	0.003151	0.000377	0.9918	73-60
12		3-methyl-butanoic acid (isovaleric acid)	A	1642	200–1000	0.008329	0.000383	0.9925	87-60
13		Pentanoic acid (valeric acid)	A	1711	200–1000	0.012637	0.000466	0.9932	73-60
14		Hexanoic acid (caproic acid)	A	1818	204–816	0.072133	0.000505	0.9981	73-60
15		Octanoic acid (capric acid)	A	2028	50–804	0.035445	0.000389	0.9632	60-73-43
16		Benzoic acid	A	2380	203–812	0.071596	0.000598	0.9913	122-105-77
17		Benzeneacetic acid	A	2503	--	--	--	--	136-91
18	Alcohols	2-methyl-propanol	A	1062	500–18500	0.139634	0.000126	0.9420	74-43
19		3-methyl-butanol (isoamyl alcohol)	A	1197	500–18500	0.645746	0.000414	0.9873	70-55-42

20		3-methyl-1-pentanol	A	1308	51–815	0.004375	0.000433	0.9980	69-43
21		1-hexanol	A	1334	25–925	0.003211	0.000520	0.9990	69-56-43
22		3-ethoxy-1-propanol	B	1360	--	--	--	--	71-59-45
23		Bezeneethanol (phenylethyl alcohol)	A	1893	25–925	0.003780	0.000833	0.9993	122-91-65
25	Acetamides	N-(2-phenylethyl)acetamide	C	2557	--	--	--	--	135-91-65
26		N-(3-methylbutyl)acetamide	B	1858	50–650	– 0.006137	0.000780	0.9918	73-43-30
27	Sulfurs	2-methyltetrahydrothiophen-3-one	B	1503	--	--	--	--	114-66
28		3-methylthio-1-propanol	A	1696	50–650	– 0.008255	0.000594	0.9939	106-61-47
24	Phenolics	2,6-dimethoxyphenol (syringol)	A	2231	50–650	0.001252	0.000985	0.9983	154-139-111
29	Carbonyls	Butyrolactone	B	1601	--	--	--	--	86-56-42
30		2-octanone	A	1267	50–793	0.006023	0.000577	0.9953	58-43
31		Benzaldehyde	A	1496	199–794	0.094028	0.000940	0.9950	105-77-51

[a] The reliability of the identification proposal is indicated by the following: A – mass spectrum, retention time, and Linear Retention Index (LRI) agreed with standards; B – mass spectrum agreed with Nist and LRI agreed with literature data; C – mass spectrum agreed with first indication in Nist virtual library (LRI not available in the searched literature). [b] LRI value calculated for the SUPELCO-megawax column. [c] No calibration curve.
